# Rethinking the role of the pretend play mode in mentalisation-focused psychoanalytic child therapy for adopted and foster children with attachment trauma: from a pre-mentalising mode to a developmental achievement and a milestone on the trajectory towards mentalisation

**DOI:** 10.3389/fpsyg.2026.1695144

**Published:** 2026-02-24

**Authors:** Patrick Meurs, Dennis Schäfer, Stefanie Hesemans, Dilan Çınar, Constanze Christina Rickmeyer, Felicitas Hug, Tamara Fischmann, Terry Meyer, Judith Lebiger-Vogel

**Affiliations:** 1 Sigmund-Freud-Institut, Frankfurt, Germany; 2 Katholieke Universiteit Leuven, Leuven, Belgium; 3 Odisee vzw Kenniscentrum Hoger Instituut voor Gezinswetenschappen, Brussels, Belgium; 4 Universität Kassel, Kassel, Germany; 5 Ziekenhuis Oost-Limburg, Genk, Belgium; 6 Group Practice and Therapy Centre ‘Het Kompas’, Schilde, Belgium; 7 Private Psychoanalytic Psychotherapy Practice, Frankfurt Region, Germany; 8 Internationale Psychoanalytische Universität Berlin, Berlin, Germany; 9 Heinrich-Boll-Stiftung, Berlin, Germany; 10 Frankfurt University of Applied Sciences, Frankfurt, Germany

**Keywords:** adopted and foster children, attachment trauma, complex relational trauma, mentalisation, pretend mode, pretend play, therapeutic use of metaphors, trauma-sensitive psychoanalytical child therapy

## Abstract

Pretend play is considered to be of great importance in both normal and problematic child development. However, the developmental and therapeutic value of pretend play has perhaps been somewhat overshadowed by the classification of the pretend mode as a non-mentalising mode, a perspective from literature on mentalisation-based psychotherapy with personality disordered adults. In this article, we highlight this difference in emphasis. After having situated this subtle and not seldom unacknowledged difference in meaning surrounding the concept of pretend mode, we focus on psychodynamic child psychotherapy for attachment-traumatised children. Early experiences of traumatic breakdowns in attachment can severely limit a child’s ability to use creative imagination in pretend play. To help the traumatised child make use of the growth opportunities of pretend play, a psychodynamic child therapist is needed who can use metaphors to integrate in an ‘as-if’ language transference-countertransference elements in their interventions, thereby creating opportunities for the child to express previously hidden trauma-related aspects. The aims of this paper are to (1) consider conceptual ambiguity of the concept ‘pretend (play) mode’ in the mentalisation literature; (2) discuss the recent diagnosis of complex relational (attachment) trauma; and (3) elucidate the role of pretend play in the psychotherapeutic process of a complexly traumatised foster child who leaves the psychic equivalent mode behind on his therapeutic journey towards moments of mentalisation.

## Introduction on the conceptual ambiguity of the pretend (play) mode

This article discusses the use of psychoanalytic, mentalisation-focused play therapy in addressing relationship difficulties resulting from complex relational trauma and cumulative losses in early attachment relationships. More specifically, it explores the unique role of pretend play in enhancing a child’s mentalisation skills, as well as the particular therapeutic techniques required to facilitate their transition into pretend play.

### Normal early development of pretend play differs from the later non-mentalising pretend mode

Experiences of early relational trauma and cumulative losses often involve temporarily interrupted or permanently severed attachment relationships with primary caregivers, a situation frequently encountered in the life stories of adopted and foster children. In the research literature, these children are referred to as being attachment-traumatised (Schore, 2009; Allen, 2013; Brisch, 2016 [1999]), developmentally trauma-disordered (van der Kolk, 2009), or suffering from complex relational trauma (Cook et al., 2005; Brewin et al., 2017). Some authors also describe these children as children with *multiple* complex trauma, a concept used to describe the repeated or sequential traumatic injury of children in their relationship to successive attachment figures and of the need for safety and belonging in primary and subsequent relationships with carers in foster homes and foster families (Vliegen et al., 2022).

Children with attachment- or complex relational trauma often experience serious difficulties with pretend play at the time of referral and in the initial stages of psychotherapy (Farnfield, 2025). Without therapy, they either never develop the ability to engage in pretend play or lose it rapidly and almost completely in the face of stress, conflict, and real or imagined threatening experiences (Boston and Szur, 1983). The development of pretend play capacities in a mentalisation-based approach enables these children to transition from the psychic equivalence and teleological modes of functioning, towards a more integrated mode of functioning, from pre-mentalising modes to mentalisation (Vliegen, 2025). Pretend mode is then seen less as a form of non-mentalising; it is rather a precursor to or developmental phase-specific forerunner or expression of unfolding affect regulation and mentalisation.

Therapists may experience moments of confusion when attempting to understand the role of pretend play, often described in MBT literature as pre-, pseudo-, or non-mentalisation, notwithstanding that it is also a crucial route to therapeutic progress. This contradiction suggests a need for clarification—a question that some authors have only recently begun to address in the last 5–10 years.

“Although the concept of pretend mode was first introduced in a developmental context, as a clinical term it has primarily been discussed in the context of adult and adolescent psychotherapy. (…) Pretend mode is a valuable clinical concept for therapists working with school-age children, but it’s use in this context needs some clarification” (Muller and Midgley, 2020, p. 16).

Mainstream MBT literature on adult patients has described three forms of non-mentalisation since the 1990s: the psychic equivalence mode, the teleological mode, and the pretend mode. These are considered to be defensive mental functioning operations against the emotional distress that mentalisation—the ability to understand one’s own and others’ psychological states—can cause (Target and Fonagy, 1996). These authors describe ‘pretend mode’ as a pre-mentalising state typical of childhood that can become manifest in problematic ways in later life, particularly in cases of personality disorder. In adult patients, the pretend mode is a way of disconnecting mental states from painful inner or outer reality in order to escape the difficult emotional consequences of that reality. Allen et al. (2008) described pretend mode as a mental state that is not flexibly connected to reality, a way of functioning psychologically that involves distortion and rigidity.

A few years earlier, Bateman and Fonagy (2004) had already described how pretend mode in adults with borderline issues can manifest itself as intellectualisation, rationalisation, and inauthentic mental processes. The individual’s mental world becomes detached from reality and appears more real than reality itself. Thoughts no longer bridge the gap between the inner world and external reality. These patients appear to mentalise and act or speak as if they mentalise—hence the term ‘pretend’—and give the impression of understanding each other’s perspective. However, as a listener, one does not really grasp the essence of what is being said; something seems to be missing: in the pretend non-mentalising mode no flexible transformation or interaction between internal and external reality can take place. Cappelen and Stänicke (2022) refer to this as “playing outside reality” rather than “playing with reality”, indicating a defensive pretend mode rather than one that is open to reality and transformation (i.e., a transitional and imaginative pretend mode). In the defensive pretend mode it seems as if the patient is mentalising, pretending to be able to speak in mental concepts. This non-mentalising “psychobabble” remains far from being in an authentic contact with the self and the other.

This form of non-mentalisation has also been associated with pseudo-mentalisation (Esposito et al., 2022) or hyper-mentalisation (McLaren et al., 2022). Although it sounds mental, it does not allow for change in therapy (Thompson and Tuch, 2014). What is lacking in the “pretend mode outside reality” (Cappelen and Stänicke, 2022) is the enchantment and liveliness present in authentic pretend play, as seen in vital children. Although children’s creative play takes place in a space that is separate or shielded from reality, it is essentially “playing with reality” (Winnicott, 1971). Shielding and protecting the play world does not mean that play is taking place outside of reality. In pretend play in childhood, both internal and external realities may influence the play script and may resonate in the play figures and scenes. And after playing, the child can also return from his make believe world to a socially shared reality (Jenkinson, 2001). This flexible relationship is lacking in the descriptions of the pretend mode in MBT literature on adult patients with personality psychopathology. In order to clarify this meaning deficiency surrounding the concept of pretend mode in MBT literature, the complex relationship between play and reality must be properly understood. This relationship is different in pretend play in childhood, compared to the pretend mode in adults with personality disorders. No one within MBT literature will fail to recognize this; (Anna Freud Centre, 2013; Fonagy and Target, 1996a and b; Fonagy and Target, 2000; Fonagy and Target, 2007) however, our point is that the differences in the concept of “pretend” have too rarely been the subject of in-depth reflection and conceptual clarification.

### Pretend play and pretend mode within a transitional space perspective

According to the developmental theory of Fonagy et al. (2002), a differentiation occurs between the internal and external worlds between the ages of two and five, before which the inner world is equated with the external world. A key feature of psychological equivalence is that anything in the mind must also exist outside of it, with no possibility of taking a different perspective.

When pretend play emerges, this action-based mode of the psychological equivalence is replaced by imaginative mental activity. It is in the transitional space between the inner and outer worlds that a path to mentalisation opens up. Through playing with reality within the pretend or make believe world of imagination, the child can differentiate between thoughts, mental representations, and fantasies on the one hand and actual realities on the other hand. For pretend play to develop, the reality of inner and external worlds must be kept at a distance, and, in between them a transitional space is needed where pretend play can unfold. At the same time, the inner and outer realities can influence the imagination of children’s play. Yet, in psychotherapy, we can observe that some children may lose themselves in defensive fantasy, in order to avoid emotional pain. Reality, for example for traumatized children, may be so harsh that they cannot connect with either their inner emotional turmoil over being abandoned and left behind or the threatening and traumatic external world.

Retreating into the psychic equivalent mode or in defensive fantasy is a non-mentalising strategy that serves not to engage with reality, but to keep it at a distance. Nonmentalising children use a language that may resemble talking about mental states or may seem like pretend play, but without dynamic or transformative links to the wounded inner reality or the anxiety-inducing external reality. This state of mind is not uncommon in patients who have learned to regulate their feelings outside of any relational context. Colombi (2010) referred to this form of pretend play as a flight into fantasy or into trauma play rather than as an imaginative play. It is a defensive strategy resulting from the loss of hope in object relations, leading the child to enclose themselves in an attempt to be self-sufficient, which hinders further mental development and creative growth-enhancing interactions. The aim in child therapy is to help the child gradually abandon this withdrawal into fantasy and to come to an authentic, nourishing relationality in the therapeutic encounter. The therapeutic challenge lies in reaching such patients and helping them connect with their genuine feelings, rather than allowing them to retreat into a private fantasy world or in self-constructed mental theories. What also should be avoided in therapy is joining them in this world of pseudo-mentalisation.

According to Muller and Midgley (2020), when relational trauma occurs in early life, separating internal states from the external world, thereby shielding the inner world behind a thick wall, is the only means of survival. There is no potential transitional space where these two worlds can creatively interact, especially when the trauma occurred before language development, during a period of which the child has no conscious memories. These patients cannot express why they are so restless and hypervigilant, so helping them to develop, within the therapeutic space, towards imaginative pretend play is a big therapeutic goal.

Until then, they remain in the equivalent mode and either do not engage in pretend play or, if they do, the play is too real, too chaotic, too wild, too repetitive or too disaffected. The therapist must challenge this kind of pretence when this mode provides grandiose and self-serving descriptions without addressing issues such as insecurity, dependency, helplessness or anxiety. This involves escaping a defensive fantasy world that ‘walls out’ or ‘caves in’ the patient. In contrast, imagination in make-believe play brings the true self into contact with the outside world, requiring moments of vulnerability, openness, and authenticity (Braman, 2023). This takes place in a transitional space that is both well-defined, protected and open to communication. It involves the ability to make use of the structure and safety provided by the presence of the therapist in a predictable playroom. However, as the clinical vignettes of a young child with attachment trauma will show, it takes time before the child engages in pretend play in the presence of the therapist. It requires special skills on the part of the therapist to bring about a breakout from the psychic equivalent and the defensive pretend mode.

## The concepts complex trauma, attachment trauma, developmental trauma and their implications for foster relationships

### Attachment trauma impacts on parental reflective functioning and child development in foster families

The early attachment histories of adopted and foster children are often affected by traumatic experiences in their relationships with primary caregivers. These early experiences of danger include unpredictability, neglect, maltreatment, and physical and psychological abuse, and can result in temporary or permanent breakdowns in relationships due to protective measures such as taking children into care and outplacing them in foster homes or families. Experiences of breakdown of early attachment can result in long-term attachment trauma (van der Kolk, 2005; Allen, 2013; Brisch, 2016 [1999]; Schore, 2017) or developmental trauma disorder (Osofsky, 2009).

Some authors refer to these forms of trauma as Type III trauma^1^ (Solomon and Heide, 1999). In unfavourable cases, these traumatic experiences are repeated and intensified when affected children are repeatedly placed in foster care and their attempts to build trust and form attachments are repeatedly interrupted. When new attachment relationships are formed with adoptive or foster parents, the traumatic primary attachment history can be reactivated, relived and repeated by the child (Streeck-Fischer, 2014[2006]). Driven by a need for security and a fear of a repetition of traumatic abandonment, some children test the robustness of their new potential attachment figures and relationships by provoking rejection, while others withdraw and appear emotionally unreachable. The fear of repetition of traumatic experiences puts pressure on children’s ability for pretend play and for emotion regulation and mentalisation, often leading to a breakdown in the development of these skills and making it harder to treat children with complex trauma (Bervoets et al., 2022). Anxiety about abandonment and separation is then seen by these children as an unavoidable reality that cannot be changed, either internally or externally. In such cases, the new attachment relationships within a foster or adoptive family are overshadowed by fear and often unbridled anger. Adoptive and foster parents want to provide their child with a positive environment and corrective relationship experiences. However, they are confronted with the child’s negative relationship expectations, which challenge their ability to provide care and attachment, and put their mentalisation ability and parental reflective functioning (Malcorps et al., 2021; Malcorps et al., 2022; Malcorps et al., 2023) to the test.

### Diagnosis of early relational trauma

The 10th version of the International Classification of Diseases (ICD-10; World Health Organization, 1992) was the first to include the diagnosis of complex trauma in the form of complex post-traumatic stress disorder (C-PTSD) in adults. This diagnosis enables the diverse and delayed (often life-long) effects of traumatic attachment relationships experienced in early childhood to be recognised and considered in adult patients. Its inclusion in the ICD-10 has led to a growing focus on the various long-term psychological consequences of early childhood relational trauma in adulthood (Herman, 1992). In the ICD-11, the C-PTSD diagnosis has been extended to include children and adolescents (World Health Organization, 2018; Cloître, 2020). This means that the long-term consequences of early relational trauma are recognised in key areas of child and adolescent development, such as affect dysregulation, impaired mentalisation, deficits in self-esteem and relationships, identity instability, and an inability to express one’s life story and everyday experiences in narrative form or in other forms of symbolisation. These symptoms and deficits in crucial developmental domains can hinder the formation of new attachment relationships in foster care and adoption contexts (Karatzias et al., 2019; Elliott et al., 2020). The repeatedly observed dynamic of rejection and relationship-breaking behaviour, or emotional withdrawal and aloofness out of a need for protection, can drive foster and adoptive parents, teachers, psychotherapists and other caregivers to despair (Vliegen et al., 2021).

In our therapeutic practice, we often observe how early trauma is carried into the therapeutic relationship. Transference offers us the opportunity to address these issues therapeutically, but it also presents therapists with significant challenges similar to those that close relatives experience (Freud, 1974; Ekstein, 1977; Chethik, 1989; Vliegen et al., 2023). How should therapists deal with the impending breakdown of the therapeutic relationship, withdrawal from it, and the re-enactment of experiences of punishment, abandonment or even physical or psychological abuse? How do therapists deal with feelings of helplessness when they are unable to reach these children, or with feelings of guilt and shame when these children make it clear that the care and help offered is not enough? How can therapists utilise aspects of their countertransference to support the therapeutic process of their clients who have experienced complex trauma? We will address this question in more detail in this text when we discuss in the next two parts of the text interventions that facilitate a child’s transition from a psychic equivalence mode into an imaginative pretend play mode.

In recent years, neuropsychophysiological (van der Kolk, 2016) and neuropsychoanalytic (Hug et al., 2022) research has revealed another perspective that helps us to understand why these symptoms of attachment trauma persist. Early complex trauma has long-term consequences not only for internal working models of attachment but also for sensory-neuro-psychophysiological response patterns. The hypothalamic–pituitary–adrenal (HPA) axis plays an important role as a complex endocrine regulatory circuit. It mediates the humoral response to stress-inducing stimuli, and under excessive stress—for example, in stressful situations or relationships—it can become dysregulated, with negative consequences for tension and emotion regulation, as well as cognitive processing. Dysregulation of the HPA axis has been associated with various disorders and impairments in memory and executive functioning (Wingenfeld and Wolf, 2011).

Internal working models of attachment and parameters of biological-neurological regulatory systems are permanently shaped by early trauma and can interact with inherited psychological vulnerabilities and/or certain personality traits, leading to trauma reinforcement and to traumatic bonding at a later moment, for example in foster care. Approximately 30% of children who have experienced attachment trauma suffer from severe socio-emotional impairment (Ensink et al., 2015), but this can be alleviated through new attachment opportunities in foster or adoptive families, as well as trauma-sensitive therapeutic intervention (Vliegen et al., 2023). It is these 30% of foster and adoptive children who are most likely to be referred to our therapeutic services.

Symptoms of complex post-traumatic stress disorder can include reliving traumatic situations, nightmares, hyperactivity, mood swings, fear of loss, emotional aloofness and self-induced breakdowns in relationships. Children who suffer from these symptoms sometimes labelled as ‘attachment-disordered’ because they behave egocentrically or self-protectively in relationships, using them functionally with little sense of their own or others’ personal boundaries and with moments of severe lack of empathy (Tang et al., 2018; Vliegen et al., 2023). New attachment relationships in a foster or adoptive family bring demands and expectations that can be overwhelming for these children, who tend to exhibit non-mentalising, reflexive responses such as fight, flight, freeze or fawning, which in turn increase the likelihood of emotional and/or behavioural problems (van der Kolk, 2016).

## Clinical illustration: psychodynamic psychotherapy with an attachment-traumatised latency child

### Biographical background of Jan

Nine-year-old Jan,^2^ a foster child, experienced a series of emotionally traumatic situations in his early relationships with his parents. There were frequent arguments and conflicts between them, and they eventually divorced. Jan had to endure his parents taking out their helplessness and anger about the divorce on him. They often rushed him off to stay with his grandparents, leaving him uncertain whether or when he would see his parents again. During these separations, Jan developed a positive attachment to his devoted and caring grandparents, but this was severely challenged by his parents’ unpredictable behaviour. When Jan was 7 years old, his grandparents asked for him to be placed with a foster family because they were overwhelmed by the care situation after several years of intensive foster care for Jan.

This experience had a serious impact on Jan’s inner world and social functioning; at referral, he was shy, insecure and tense. He seemed very concerned with doing everything ‘right’. For example, he repeatedly expressed his fear of losing the approval or availability of others because he believed he did not deserve it. Jan was a boy who found it difficult to let others get close to him. When confronted with his own intense feelings, whether positive or negative, he froze completely. He avoided talking about his life story, his feelings and his family.

The diagnostic material shows that Jan associated his inner perceptions of caregivers with unpredictable threats and dangers. In the ‘Family in Animals’ test (Brem-Gräser, 1992 [1957]), he imagined his family members as people who ‘suddenly bite hard’, ‘have sharp teeth’, and ‘sometimes scratch unexpectedly’. His drawings and stories revealed the unpredictability of human relationships, with threatening figures suddenly appearing in drawings that previously had a completely different emotional tone. Some of the stories in the Attachment Story Completion Task (Bretherton et al., 1990) contained positive images of individuals who engage with and praise the child. Jan explicitly and exclusively associated these positive images of care with the caregivers in his foster family. Yet, in moments of conflict and tension, these positive relationship images were overlaid by strongly negative images and relationship patterns, rendering the positive images ineffective.

Jan said that, in moments of fear, ‘his head gets confused’ and ‘there is no one there to help him’. Feelings of anger and sadness reinforce each other: when he is annoyed by others, Jan first becomes very sad and then very angry, or he withdraws from social contact. This withdrawal initially calms him down, but he soon starts to feel cut off from all contact, abandoned by others, and so alone that he panics. These moments of emotional withdrawal or psychic retreat (Steiner, 1993) serve to ‘regulate’ the intensity of the feelings associated with the fear of being abandoned, making these feelings somewhat more bearable. At the same time, withdrawal leads to loneliness and intense panic. Once he has lost contact, he does not know how to re-establish it except through problematic behaviours that attract attention but also provoke rejection, thus perpetuating the dreaded feeling of being left alone.

### Examples of the complexity of trauma-sensitive therapeutic work with Jan

Jan was referred to the therapy center^3^ for various reasons: he seriously questioned his relationship with his foster parents and made them feel that they could never live up to his expectations. He believed that they were the reason why he could not stay with his biological parents or grandparents. In addition, over the past period, he has begun to display verbal and physical aggression towards his foster parents. The foster parents increasingly felt that they were getting bogged down in this struggle with Jan and that Jan’s trust in what they offer him was dwindling. Above all, the feeling that they could not offer Jan what he really longed for led to disappointment and anger as well as feelings of guilt and shame on the part of the foster parents.

Contacts with his foster parents and, later on, with his therapist were also frequently overshadowed by behaviours that raised questions about whether they were driven by internal working models associated with Jan’s early, dysfunctional, and broken relationship experiences: mistrust, vigilance, and disbelief that the other person could give him anything he could use in his life. Epistemic trust is something Jan lacked: he had no feeling that social information and the relational offerings of others can be trusted or might be meaningful to him.

What we want to highlight in the following excerpts from Jan’s therapy, are the relationship difficulties that he also brought into it: How he initially tried to keep everything problematic out of the contact by acting in an artificial and overly adapted manner, then expressed his ambivalence by pushing the therapist away, subsequently began to express the first symbolisations of this problem in the form of pretend play fragments and in a story he told the therapist, and finally communicated a desire for a different kind of contact with her.

Jan began psychoanalytic child psychotherapy, in the context of the Belgian research project Leuven Adoption Study (LAS).^4^ He attended therapy for 1 h each week, from the second year of therapy on, 2 h each week. He had been placed in his foster family 2 years earlier. In the relationship with his birth parents, he had repeatedly experienced painful separation and loss, and had been punished in sadistic ways. Initially, Jan found it difficult to open up emotionally and establish a connection with his therapist or foster parents, so it took time for a stable relationship to develop. He appeared cautious and withdrawn, and at times completely shut down. He was extremely sensitive to even the slightest sign of separation, reacting with hurt, anger and despair. The therapist got the impression that Jan was firmly convinced that any contact would be lost if he revealed his emotions and showed vulnerability in his need for recognition. His foster parents reported that Jan’s behaviour often provokes rejection and punishment, leading to heated situations when they set limits on his behaviour. He then becomes angry and withdraws, feeling hurt. This made them feel guilty. Jan often asked them if they loved him and if he belonged.

His latent restlessness and ambivalence in relationships came to a head in the tenth therapy session. As the therapist and Jan entered the play therapy room, he suddenly ran to the window, opened it and shouted to a passer-by on the street, “She’s abusing me! Help me! She’s hitting me! Please help me! She’s hurting me! Quick, help me!” The therapist was taken aback, but quickly regained her composure. She clearly instructed him to close the window and asked him what had happened. Jan screamed that she cannot help him and called her stupid and not a real psychologist. Such serious accusations often arise in therapy with attachment traumatised children, as we will also see in one of the next clinical vignettes.^5^

**Clinical vignette 1:** ‘I do not want to do anything wrong under any circumstances, so as not to jeopardise the relationship.’

At the beginning of therapy, Jan seemed intent on behaving perfectly. He apologised in advance for any perceived mistakes. In the fifth session, he and the therapist played Jenga together. They built a tower out of blocks, taking turns to remove a block from the tower and place it on top. The longer the game lasts, the more unstable the tower becomes. Whoever causes it to collapse loses the game.

*(It took Jan a while to pull a block out of the tower.) J: ‘Hmm, that’s difficult. Sorry it’s taking so long.’*

*Therapist (T): ‘Take your time; it’s not easy.’*

*J: ‘Sorry. (He searches until he finds a spot that seems easier.) Finally!”*

*T: ‘Let us see. Now, I’ll put this here. Then it’s your turn again.’*

*J: ‘I’m sorry I’m so slow. I’m really sorry if I knock the tower over. Sorry!’*

During the initial phase of therapy, the therapist realised that Jan apologised for almost all behaviour. For example, he expected the therapist to find it annoying if she had to wait for him, even for a moment, while playing, either because he needed time to place a building block correctly or because he knocked the tower over. Very early on in therapy, he mentioned that he feels that others find it annoying to listen to him or play with him. Every glance or reaction from the therapist, his foster parents or his friends at school seemed to confirm his negative expectations and reinforced his apologetic behaviour. He repeatedly tried to control his fear of not being good enough by apologising for things he had not done or that had not happened yet.

Always being on guard and apologising, Jan provoked exactly what he wanted to avoid: irritation. The therapist paid him a lot of attention, but this frightened him. After each session, he sought reassurance that he could return for another session; he craved the therapeutic contact, yet this desire also made him uneasy. For a long time, Jan did not dare take the lead and decide what to play for fear of the therapist’s disapproval.

**Clinical vignette 2:** ‘I push you away so you cannot push me away.’

Jan could become very angry at any moment, making serious accusations against the therapist and then withdrawing from the relationship. This, however, led to feelings of loneliness, which Jan found hard to tolerate. In these situations, the therapist tried very hard to help Jan, but Jan could not neither accept this attention nor sort out his own feelings. He was ‘hard to reach’ at such times. Jan’s foster parents also reported this difficulty.

*During the 12th session, when Jan appeared emotionally blocked and did not know what to do, the therapist asked how she could help.*

*Jan replied,* “You can’t help me!”

*T: ‘Can anyone or anything else help you?’*

*J:* “Yes, a special psychologist, a real one...”

*T: ‘A special psychologist? Couldn’t that be me?’*

*J:* “No, I want someone who is really special.”

*T: ‘What would that be?’*

*J: (sighing deeply several times, shrugging his shoulders, then loudly) ‘No! You’re not! You cannot! No! No! Shut up!”*

*T: ‘You feel that I do not understand you, even though I’m trying?’*

*J: ‘You do not have a degree, yet you write everything for the juvenile court and report to the judge. You’re stupid, stupid, an absolute idiot.’*

We see in the second vignette the clear breakthrough of negative images and expectations, as well as the devaluation of the therapist. This happened not so long after the incident with opening the window and calling for help from a random passer-by. The therapist sensed Jan’s deep despair behind his anger and wanted to help him and bring him out of his frustrated withdrawal. In a psychoanalytic therapy model, the conflict perspective, in the form of an inner conflict between desire and fear—in this case, the desire for contact and the fear of losing it, or the fear of not getting what one needs from the other person—is central. This perspective helped the therapist to better understand Jan’s conviction that contact with her could not offer him anything, even though he needed it so badly. It also helped the therapist to counteract Jan’s relationship patterns—his accusations, pre-emptive apologies and fears, such as not being allowed to come to the next session—in a containing manner.

**Clinical vignette 3:** ‘I shut myself off so you cannot reject me.’

Phases of ambivalence were often followed by emotional withdrawal from the therapeutic relationship in an attempt to minimise the intensity of fears related to the trauma of abandonment. This is demonstrated in an excerpt from the 36th session.

*During the previous session, Jan drew a picture of his home, but then threw it in the bin because he thought it was bad. Now, he wanted to draw trees, but he did not succeed.* “Everything looks ugly,” *he says.*

*T: ‘You would like to paint trees, but you are unsure whether it will turn out well.’*

*J: ‘How am I supposed to do that?’ (Covering his eyes with his hands, he turns away from the therapist in anger.)*

*T: ‘Not being able to paint makes you angry and sad at the same time, so you want to hide away.’*

*(In response to Jan’s prolonged silence after her intervention.)* “Would you like me to wait for you, or maybe sit a little further away, until you feel ready to talk to me again?”

*J:* “I don’t want to come anymore, never again! Never, never!”

*Jan remained standing with his back to the therapist until the end of the session, holding his hands over his ears and eyes. He did not say a word and did not want to listen to the therapist.*

In our assessment, Jan found it difficult to accept the therapist’s offer of help because he struggled with inner images associated with intense fear and the terrible experience of being left alone and abandoned in moments of tension and helplessness. He withdrew from contact to keep these difficult emotions, thoughts and inner relationship images at bay. As a result, he lost his connection to both the therapist and his inner emotional world.

Due to early attachment trauma, Jan struggled with the feeling that rejection is inevitable. This was accompanied by feelings of worthlessness, which manifested as negative self-perceptions and beliefs: ‘I am worthless’ or ‘If I show who I really am, they will dislike me anyway.’ These thoughts and fears triggered a strong defence mechanism, causing Jan to withdraw completely from contact. While this withdrawal could provide short-term stress relief, it also led to loneliness and relationship difficulties, exacerbating the stress and social anxiety he experiences. In literature on attachment trauma, this is described as the *biopsychosocial trap* (van der Kolk, 2016; Vliegen et al., 2023): biological tensions lead to negative psychological representations and social withdrawal, which increase biological tensions and have a negative impact on future relationship experiences over time. Consequently, Jan became increasingly trapped in a vicious cycle of circular reasoning. This quasi-automatic process is discussed in more detail in clinical vignette 5. But before we get to that vignette, we need to discuss a breakthrough in therapy in vignette 4.

**Clinical vignette 4:** ‘The safe door slams shut. Now I’m trapped, like in a cage.’

During supervision, the therapist used the metaphor of a door that suddenly slams shut and cannot be opened even a crack, to describe Jan’s reticence. She felt that she could not reach him, and that he could not escape his isolation. In the 54th session, she used this metaphor which was deduced from her countertransference, to help Jan understand what is happening in therapy. Jan immediately understood what was meant and how to apply it to himself and was able to communicate something about his relationship needs.

*T: ‘I was still thinking about the last session, Jan. In difficult moments between us, it’s as if a door were slamming shut. I cannot get in, and you cannot get out. I cannot see what’s happening behind it. I cannot open the door, not even a crack. No matter how hard I pull on it, nothing seems to help open the door in those moments.”*

*J: (agreeing) ‘But sometimes the door is open.’*

*T: ‘You feel that the door is open sometimes. Maybe we can figure out together how to keep the door open in those moments?’*

*J: ‘I do not know how to get the door to open again.’*

*T: ‘Sometimes it seems to close again right away. ‘Poof’, closed, and then there’s no gap left.’*

*J: ‘Then I’m trapped in a cage, … like with Mum.’*

*T: ‘You say you are trapped in a cage? And the cage reminds you of your mum?”*

*J: (Nodding.) “Yes, a cage...” (with tears in his eyes): ‘It’s like with Mum.’*

*T: ‘Those moments remind you of your mum.’*

*J: (screaming and covering his eyes with his hands): “Go away! Go away! Get out! Stop! Leave me alone! ... This is what my mum was saying then.”*

*T: (After Jan hesitantly takes his hands down) ‘Now I can see you, Jan, as if the door were open a crack and you were no longer locked in the cage.’*

*Later in the session, he said that he feels angry and sad in the cage. He feels that everything is ‘completely falling apart’ and he does not know how to ‘put the pieces of the puzzle back together’.*

*J: ‘When the door is closed, it’s already too late’.*

*T: ‘We have to find out how to prevent the cage door from suddenly slamming shut, and how to maintain important contacts.’*

Unlike before, Jan could now see actively breaking off contact as something his mother did. He could distance himself from this attitude and, with the help of therapy, start to develop a different relational pattern. The therapist’s countertransference image of a door slamming shut and preventing people from reaching Jan enabled him to recognise this behaviour in himself and situate it as something he learned from his biological mother. Through the “as if” quality of the therapist’s statement (“it is as if a door is slamming shut”), Jan learned that what is being discussed does not necessarily have to be true just because he fears it. This “as if” quality leaves room for further steps on the developmental path towards mentalisation, which can be regarded as a developmental milestone and achievement.

**Clinical vignette 5:** ‘No longer completely alone in the cage’; one can begin to turn towards another on a bodily and affective level, with grief that has been unbearable and unthinkable until now.

As the therapy progressed, Jan gradually became more aware of when the cage door was about to close. He then took some time for himself and signaled to the therapist when he was ready to continue talking or playing. Sometimes, however, this did not work, and the door slammed shut again. Yet, during these moments, the therapist sensed that something had changed, which made it somewhat easier to stay in contact with Jan.

*In the 60th session, Jan said that he was tired, hungry and had a headache. When the therapist tried to link this to an incident at school or to something he had mentioned earlier in the session, Jan reacted angrily.*

*J: (shouting): “That has nothing to do with it! The pain is also there in the morning, and I’m not stressed then!’*

*T: ‘Are you angry because I’m asking you about your headaches?’*

*J: ‘Not at all!’ (He turned away from the therapist and started crying loudly.) (Although he initially blamed the therapist and expressed his anger, he looked at the therapist with a sad expression on his face. Allowing this sadness to surface and communicating and dealing with it is new for Jan.)*

The fact that he could express in a former session, in a figurative and metaphorical way, that withdrawing from relationships or breaking off contact carries the risk of him becoming trapped in a closed space (a cage), and that he could contemplate this without becoming consumed by endless accusations about the therapist’s worthlessness, offered hope for therapeutic change. This meant that Jan could recognise the meaning of his behaviour and start to express these insights while talking, drawing or playing, rather than withdrawing or acting out as the only ways to escape the pain of his relationship traumas. However, this therapeutic progress could be undermined by any unbearable stress or conflict in his relationships.^6^ In this phase of the therapy, he was not yet very good at talking about these difficulties from his own perspective (the perspective of the self). It was indeed much more difficult for Jan to clarify this issue when it was addressed in terms of the self than when he could remain within the metaphor to start to explore it, as was the case in vignette 4. Such metaphorical communication has an ‘as-if’ quality, as the therapist herself noted: ‘As if a door were closing.’ This enabled Jan to explore and express his feelings. This form of communication marked a turning point in therapy, opening the way to mentalisation in a more integrated way. The change that had occurred in the first year of therapy became clear in the sixtieth session, when he no longer shielded himself from experiencing his sadness. He finally looked at the therapist, an important opening in the (bodily) contact that had not yet happened before, in moments of yearning, despair and tension. This change brought hope that such feelings could now be discussed and explored more often, and that a beginning of addressing and working through the pain of the attachment trauma was now possible.^7^

## Complex trauma-specific therapeutic skills: translating countertransferential aspects into metaphors as a way to introduce the child in the pretend play mode

The final section of this article illustrates how Jan’s capacity for reflection evolved during therapy, progressing from being confined within the biopsychosocial trap to developing a nascent mentalisation ability, which necessitated substantial ego-support over an extended period of time. As previously mentioned, Jan found it difficult to speak in the first person, so presenting him his difficulties in the form of a metaphor was a crucial intermediate step for Jan in learning to embrace the as-if mode of thinking and, in a next step, to speak from a first-person perspective.

But let us first look back in the therapy process. Before entering the therapy room for Jan’s first meeting with the therapist, Jan’s foster mother had taken the opportunity to ask the therapist a few questions in the waiting room. Jan witnessed this and became convinced that they were speaking negatively about him and were mocking him or making him look ridiculous. It was difficult to dissuade him from this belief. He insisted that he could see them mocking him. This was a challenging start to therapy, indicating a lack or defect of epistemic trust (Fonagy and Campbell, 2017). Without epistemic trust, information that could correct Jan’s belief does not reach him; aversive feelings dominate the situation and override his reflective abilities. In the psychic equivalent mode (Fonagy and Target, 1997), internal and external reality are equated. When Jan was in this state of mind, he perceived his own interpretation of events (“You talked badly about me and mocked me”) as the only possible reality.

In a later session (see Clinical Vignette 2), Jan was convinced that the therapist was unqualified and unable to help him. The child equated the therapist with his inner image of her, shaped by fearful expectations and a devaluation of the therapist: ‘She is useless; she cannot help me.’ His reaction in situations similar to those described earlier in this article—running to the window and shouting to a passer-by that the therapist is treating him badly—is another example of Jan’s attempts to alleviate his inner pain. Here, inner and outer realities were linked by the acting-out behaviour of opening the window, shouting and accusing the therapist. This is considered a teleological mode of pre-mentalisation (Fonagy and Target, 1997; Duschinsky and Foster, 2021).

In conditions of epistemic mistrust, no lasting therapeutic change can occur. Adopting a therapeutic attitude in which the therapist patiently tried to understand and verbalise Jan’s feelings and thoughts (the therapist functioning as a reflective mind; Fonagy and Target, 1997) showed the child that help can be found in relationships, promoting the development of trusting relationship representations. With the therapist’s support, Jan gradually learned to abandon one-sided interpretations of events and consider other perspectives. Although this experience did not immediately lead to a long-lasting change in attitude, it reduced tension and mistrust in the current situation. This is a prerequisite for the development of alternative relationship representations over time.

Under great stress, Jan slipped from a controlled, explicit thought process to an automatic, implicit one. At this point, evolutionary protective functions took over: fight (e.g., blaming others), flight (e.g., retreating into a shell, a cage, a ‘claustrum’ as Meltzer (1992) describes this situation), freezing and fawning (van der Kolk, 2016). These behaviours primarily correspond to the two pre-mentalising modes of teleological thinking and psychic equivalence. Learning from relationship experiences, including in the therapeutic relationship, then becomes very difficult or even impossible for patients.

In contrast to the previous two modes, the pretend mode of play (Fonagy and Target, 1997) enables one to find a situationally appropriate, metaphorical expression of one’s inner reality in therapeutic play and ultimately to understand one’s own emotional experience in a relationship with another person. In this mode, inner and outer reality are no longer equated. In the psychoanalytic literature, the ability to express inner emotional experiences metaphorically during therapeutic play is an important developmental milestone in the development of symbolisation and mentalisation capacity (Muller and Midgley, 2020).

During phases of therapeutic play but also during children’s normal playtime, the child may retreat from external reality for a moment and enter a pretend play mode in a transitional space in which the child can completely immerse himself and let his imagination run wild, perhaps made possible by the fact that others around him facilitate this “capacity to play alone in the presence of the other” (Winnicott, 1958), shield him and keep this play mode safe (Fein, 1981). In mentalisation literature, mostly concerning adult borderline patients, the pretend mode is described as a defence mechanism involving withdrawal into fantasy and omnipotence. Fantasy in this context loses its playful imaginative character and becomes defensive. It no longer serves to express the relationship with reality nor does it help the child approach reality with a richer imagination. In normal development, imagination and pretend mode enable creative engagement with the complexity of the relationship between inner and outer worlds. In pathological development, retreating into a fantasy world leads to an inability to form a reciprocal relationship with the internal or outside world. Against this background, the pretend mode is often considered a form of non-mentalisation that hinders the development of the integrated mode of mentalisation. However, what is sometimes overlooked in this view is that entering and remaining in an ‘as if’ mode in the presence of the therapist can facilitate the perception and communication of the child’s inner world of thoughts and feelings. Initially, this can be achieved in the context of pretend play with various play figures, and ideally, at a later stage, with the involvement of the child’s self, whose ability to communicate is fortified by a maturing ego.

In this pretend mode in normal development, children often engage in role-playing, taking on the characteristics of figures or characters in their imagination and fantasy play with peers or in therapeutic contexts. In this mode, they can try out different roles and experience the perspectives of self and others (Freud, 1927; Piaget, 1951; Nicolopoulou and Ilgaz, 2013; Koukourikos et al., 2021). This enables them to discover the perspectives of others and incorporate their own inner states as well as the inner states of others into their interactions. By doing so, children can find a path to change, guided by therapists who play along with them. And also outside the context of the therapy play room, pretend play enables the transition to an integrated mode of mentalisation (Fonagy and Target, 1997).

Jamrozik et al. (2016) describe how metaphoric communication in therapeutic play creates a state of mental activation and attention, allowing the child to feel addressed. For Jan, the metaphor of the cage and the locked door in the therapeutic situation established a connection between his inner and outer worlds. This metaphor is both concrete and abstract, and helped him to understand and integrate his experiences. Its novelty evoked surprise and captured his attention (Krimendahl, 1999; Bretherton, 1984). Recognising a part of himself in this metaphor had a synthesising or integrative effect (Folmo et al., 2021). Furthermore, its effectiveness stemmed from its role in the transference process: In the therapeutic relationship, Jan associated the feeling of being trapped in a cage with his relationship with his birth mother and foster parents. However, this image could only emerge and be processed meaningfully because this relationship dynamic was re-created scenically in the therapeutic relationship and could be expressed with the help of the therapist (Brandell, 2017).

This could also clarify an aspect of the meaning of Jan’s highly irritating behaviour in the tenth session: While the therapist initially interpreted the opening of the therapy room window as acting out, in hindsight, and with an understanding of the metaphor about the ‘caving in dynamics’ of the door of the cage slamming shut, the opening of the window can also be seen as a pre-symbolic expression of moving away or wanting to escape from a closed, distrustful and fearful relationship repertoire that resulted in terrible isolation. The opening of the window could then—in addition—be seen as a pre-symbolic expression of opening the cage of isolation and the trap of unbearable loneliness that are central to the relational life of complexly traumatised children.

## Conclusion: rethinking the role of pretend play and the pretend mode in child therapy

When working therapeutically with children affected by attachment trauma, it is important to consider the main themes and relationship patterns associated with this condition, as discussed in this article. These children feel and fear that they are not worthy of what others offer them. They make desperate attempts to act perfectly in order to control their negative self-image and inner representations of relationships. In order not to be left behind, they also push others away, thereby closing themselves off, existing in an empty, lonely cage. During the here described therapeutic process, the 9-year-old patient, Jan, increasingly sensed that thereby he was entering a situation reminiscent of the trauma of abandonment, which these children fear so much and want to escape from.

Jan’s case clearly showed how important it is to provide mentalisation-focused interventions, in order to prevent these children from repeatedly experiencing traumatic relationship dynamics in everyday life and in therapy. As his therapy progressed, it became clear how strongly Jan’s tendency to withdraw shaped his relationships, and, eventually, how maintaining contact in therapy supported his further development. Small changes to existing patterns can be made in therapy. They are identified, tried out and explored in sessions and can be gradually observed and discussed. Children are encouraged to try out these changes outside therapy and learn from their successes and failures. It is a hopeful process, but one that includes many moments of relapse and resistance amid the patients’ progress.

Jan’s therapy demonstrated how the introduction of the metaphor of the cage—which emerged from his transference of feelings of mistrust, abandonment and imprisonment and was interpreted by the therapist—can facilitate progress. Jan was able to accept this intervention that was presented in an ‘as-if’ mode and he related it to his relationship with his biological mother, thereby moving beyond pre-mentalising modes of functioning. Consequently, this as-if approach became a turning point, enabling the exploration of pretend play material focusing on rooms that can be closed off or be opened. The first moments of symbolic play in a pretend mode were an important change in Jan’s therapy, paving the way for him to progress towards moments of an integrated mode of mentalisation. This mode occurred when the play content and his narratives over relationship experiences in the outside world were linked.

To conclude, we can say that this entire article demonstrates that pretend mode encompasses much more than a non-mentalising state. The authors of mentalisation theory will certainly recognise this and perhaps argue that they do not refer to the pretend mode of adults as ‘pretend play’, but as ‘pretend mode’ or dysfunctional mentalisation, thereby indicating a clear difference. Nevertheless, it is confusing to see a well-known concept from the psychology of normal development applied to a phenomenon involving borderline adults who pretend mental states or experiences in an uncreative manner, without a focus on transformation, change, or therapeutic growth. Transferring an existing concept from normal developmental psychology to the context of clinical psychology and from an early developmental stage into a problematic mode of thinking in a later phase of life can be associated with the risk of inadvertently shifting the meaning of the concept.

While mentalisation theories certainly do not underestimate the value of pretend play in early childhood, they probably do not discuss sufficiently the value of the pretend mode as a gateway to mentalisation in latency children and maybe also in older patients. The therapeutic skill needed to facilitate the transition from the equivalent to the pretend mode, is the active use of the countertransference and the conversion of these countertransference aspects into metaphorical communication (Enckell, 2010). This allows the child to enter the pretend play mode in a transitional space that is opened up for them by the therapist’s intervention. Without this therapeutic skill, there is a risk that the complexly traumatised child remains stuck in psychic equivalence and teleological modes. As discussed and clarified in this article, these modes or maladaptive states of mind should not be mentioned in the same breath as the pretend mode.

## Data Availability

The original contributions presented in the study are included in the article/supplementary material, further inquiries can be directed to the corresponding author.

## Footnotes

1 Type I traumas result from overwhelming natural forces (nature made) and are usually one-off events, such as a tsunami, house fire or flood, or an accident that someone has experienced or witnessed at close range. Type II traumas result from human behaviour (man-made) and can be either a single event, such as rape or sexual assault by a stranger, or multiple events, such as long-term bullying at school or work or being ostracised by one’s family or growing up in war circumstances (Terr, 1991). Type III traumas are a subset of Type II traumas caused by humans. These refer to long-term abusive or neglectful relationships in which children are victims of their own biological parents—the first attachment figures from whom one would expect security and devotion, but who, in reality, treat the child destructively. Some people also classify these traumas as Type II, referring to Type III only when additionally the relationship with the biological parents breaks down as a result of the abuse and neglect (for example, in cases of adoption or long-term foster care). Others use the term ‘Type III’ when the human cause of the trauma can be found within the individual’s own family circle, and when this traumatogenic factor has been present for a period of time. For these authors, type III indicates that maltreatment, neglect and exclusion take place in the context of the attachment relationship where the child would expect to find security, care and love (see also: Vliegen et al., 2021).

2 All names have been anonymized and biographical details actively encrypted.

3 Therapy Center “Praxis P” of the Faculty of Psychology and Educational Sciences at KU Leuven (Belgium), where therapy is provided in the context of postgraduate training in psychoanalytic child and adolescent psychotherapy. The therapist in Jans psychotherapy was Stefanie Hesemans. Supervisor was Christine Leroy.

4 In this foster care and adoption study, the foster parents and the child themselves gave permission for the therapy material to be discussed in educational contexts and in publications. Video recordings of the therapy sessions were made, transcribed and analysed. After each session, the therapist recorded her own impressions and thoughts in a notebook. She received individual supervision for this case and participated in team discussions about other foster children undergoing therapy. The team was supported by the research leaders, Prof. Nicole Vliegen and Prof. Patrick Luyten of Catholic University of Leuven in Belgium.

5 Not all foster children who are referred exhibit the same dynamics, but the points described here are – in varying degrees – readily recognisable in other foster and adopted children. Drawing on these dynamics, Vliegen and her colleagues have written a manual for psychodynamic child therapy for complex trauma (see Vliegen et al., 2023).

6 This article describes significant therapeutic progress. However, it is common for children with complex trauma to experience serious setbacks when faced with challenging situations or transitions in life, such as starting a new school or entering adolescence. While therapeutic progress is not guaranteed to be permanent, it does offer a starting point in times of relapse, meaning that crises last for less time. Foster parents must be reminded of this perspective time and again in times of crisis.

7 The parental guidance that accompanies these therapies is a topic that would require its own article. Some recurring aspects are: realising that these children are different from what one could ever have imagined when one started caring for them; carefully considering which conflicts to engage in and which to leave alone (‘choose your battles’); never really knowing what is truth and what is fearful fantasy when listening to stories about incidents and conflicts; these children usually suffer from disharmonious developmental profiles (e.g., intelligent but socially and emotionally difficult); realizing that every new developmental step potentially brings a relapse and that parenting these atypically developing children requires much more effort; after a while in treatment the parents realise that they become attached to parental guidance sessions because there they can talk about things that they—even in foster care organization’s parent’s groups—do not dare to talk about, etc.
